# Mechanical, Hormonal and Psychological Effects of a Non-Failure Short-Term Strength Training Program in Young Tennis Players

**DOI:** 10.1515/hukin-2015-0009

**Published:** 2015-04-07

**Authors:** Jose Manuel Sarabia, Jaime Fernandez-Fernandez, Casto Juan-Recio, Hector Hernández-Davó, Tomás Urbán, Manuel Moya

**Affiliations:** 1 Sports Research Centre, Miguel Hernandez University, Elche, Spain.

**Keywords:** power output, resistance training, cortisol, mood states, youth athletes

## Abstract

This study examined the effects of a 6-week non-failure strength training program in youth tennis players. Twenty tennis players (age: 15.0 ± 1 years, body height: 170.9 ± 5.1 cm, body mass: 63.3 ± 9.1 kg) were divided into experimental and control groups. Pre and post-tests included half squats, bench press, squat jumps, countermovement-jumps and side-ball throws. Salivary cortisol samples were collected, and the Profile of Mood States questionnaire was used weekly during an anatomical adaptation period, a main training period and after a tapering week. The results showed that, after the main training period, the experimental group significantly improved (p<0.05) in mean and peak power output and in the total number of repetitions during the half-squat endurance test; mean force, power and velocity in the half-squat power output test; Profile of Mood States (in total mood disturbance between the last week of the mean training period and the tapering week); and in squat-jump and countermovement-jump height. Moreover, significant differences were found between the groups at the post-tests in the total number of repetitions, mean and peak power during the half-squat endurance test, mean velocity in the half-squat power output test, salivary cortisol concentration (baselines, first and third week of the mean training period) and in the Profile of Mood States (in fatigue subscale: first and third week of the mean training period). In conclusion, a non-failure strength training protocol improved lower-limb performance levels and produced a moderate psychophysiological impact in youth elite tennis players, suggesting that it is a suitable program to improve strength. Such training protocols do not increase the total training load of tennis players and may be recommended to improve strength.

## Introduction

Tennis involves intermittent, high-intensity efforts interspersed with periods of low-intensity activity, during which active recovery (between points) and passive periods (between changeover breaks in play) take place, over an extended period of time (i.e., in some cases > 5 h) (Fernandez-Fernandez et al., 2009). Throughout matches and practice sessions, players are constantly required to execute explosive actions (i.e., accelerations, decelerations, changes of directions, and strokes) with precision and within a very short period of time, highlighting power as a key determinant of tennis success (Fernandez-Fernandez et al., 2009; Reid and Schneiker, 2008). Therefore, the optimal design and implementation of training strategies that enhance power seem to be important for coaches and players.

The effectiveness of a strength training program depends on the application of appropriate training loads, which is related to the proper handling of training variables such as intensity, volume, and frequency, among others (Kraemer and Ratamess, 2004). Coaches and sport scientists in the field of strength training have attempted to identify proper handling of training variables to determine the training stimulus that maximises performance enhancement, although the optimal combination of such training variables is still under debate (González-Badillo et al., 2005; Izquierdo et al., 2006). It has been suggested that the main effect (i.e., neural, hypertrophic, metabolic, and hormonal responses) and subsequent adaptations to strength training partially depend on the total number of repetitions performed by an athlete (Izquierdo-Gabarren et al., 2010). In this regard, training leading to repetition failure (inability to complete a repetition in a full range of motion due to fatigue) or not leading to failure has been of interest in the past two decades (Drinkwater et al., 2005; Izquierdo et al., 2006; Rooney et al., 1994). The primary role of training leading to repetition failure has been related to the increase of the motor unit activation capacity and high stress levels to the tissues, which would increase protein synthesis in order to repair damaged muscle during the training process (Drinkwater et al., 2005; Rooney et al., 1994). Short-term training (< 9 wk) leading to repetition failure produces greater improvements in strength when compared with a non-failure training approach (Drinkwater et al., 2005; Rooney et al., 1994). However, other studies have reported that training to failure results in a small effect and may not be necessary for optimal strength gains, because the incurred fatigue reduces the force and velocity a muscle can generate (Folland et al., 2002; Legaz-Arrese et al., 2007; Sanborn et al., 2000).

In sports requiring maximum power, strength exercises should be performed explosively, reaching the maximum velocity allowed by the load used (Jones et al., 1999; Munn et al., 2005). A reduction by more than 5–10% of the execution velocity could deflect the training effect towards endurance, promoting non-desired effects (i.e., stimulation of slow fibres), and not towards reaching maximum power (Fry, 2004). However, in modalities in which strength demands are not very high (i.e., recruitment of all fast fibres and depletion of PCr stores are not required), it is possible that the execution of fewer repetitions while maintaining power levels would not have great relevance. In tennis, although there has been little research to substantiate the efficacy of strength training programs for players (Reid and Schneiker, 2008), based on previously mentioned mechanical demands (i.e., power generation during strokes and movements), it seems that the development of maximum-strength levels is not required. Thus, it can be argued that the use of training programs not leading to muscular failure (i.e., programs based on the maintenance of mechanical power), and only using those repetitions that maintain maximum power, would be useful to increase the overall power demands in tennis players (Izquierdo et al., 2006; Legaz-Arrese et al., 2007; Sanborn et al., 2000).

In addition to the mechanical aspects (i.e., power output), the homeostatic hormonal changes in response to strength training have been thought to play an important role in strength development (Kraemer and Ratamess, 2005), and the acute response of several hormones (i.e., testosterone, human growth hormone and cortisol) has been suggested as a useful marker of chronic strength training stress (Kraemer and Ratamess, 2005). Salivary cortisol (SC), as a representative marker of circulating free cortisol (Hellhammer et al., 2009), has been recommended as an index of training stress in sport settings, because it avoids the stress caused by venepuncture, thus reducing artificially high values due to an anticipatory effect (Gatti and De Palo, 2011). In tennis, SC has been used to determine the acute psychophysiological stress responses during training cycles and competitive single matches (Filaire et al., 2009; Rouveix et al., 2006), although there is no information about the responses in tennis players during a strength training program. Together with the hormonal changes produced by exercise, it also seems important to measure the impact of manipulating training variables (i.e., volume, intensity) in the athletes’ mood states (Leunes and Burger, 2000). The Profile of Mood States (POMS), which reflects an individual’s mood in six primary dimensions (i.e., Depression-dejection, Tension-anxiety, Anger-hostility, Vigour-activity, Fatigue-inertia, and Confusion-bewilderment), has been widely used in sports to evaluate the psychological state of athletes (Jones et al., 2010; McNair et al., 1997). High values on the Vigour-activity scale and low values on the remaining scales are desirable for athletic performance.

Thus, the aim of this study was to analyse the effects of a short-term strength training program not leading to failure in young tennis players. Additionally, SC and mood states were also monitored in order to detect any possible relationship between performance changes (i.e., power output) and psychophysiological stress.

## Material and Methods

### Experimental Approach

**Figure 1:**
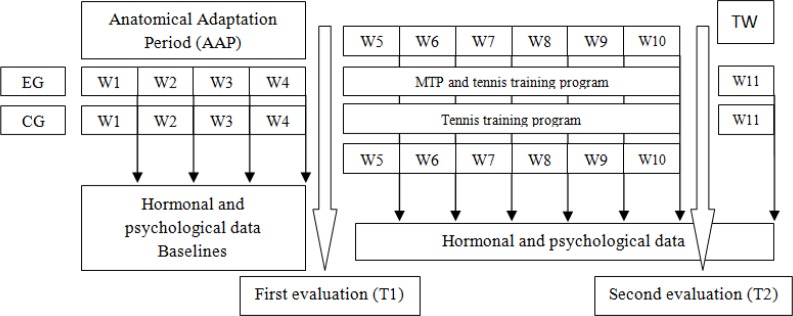
Experimental design.

A randomised, controlled and longitudinal (i.e., pretest-posttest) design was used in the present study. Before any baseline testing, all of the participants attended a laboratory for a familiarisation session to introduce the testing or training procedures, and also, to ensure that any learning effect was minimised for the baseline measures. Training was conducted during the pre-season (September to November). Pre (T1) and post-tests (T2) included: Parallel half squats, Supine bench press, Squat Jumps (SJ), Countermovement Jumps (CMJ) and Side Medicine Ball Throws. Moreover, hormonal (SC) and psychological data (POMS) were recorded once a week (Sundays). The training intervention consisted of eleven weeks divided into: four weeks for an anatomical adaptation period (AAP); six weeks for a main training program (MTP), and a tapering week (TW) (Figure 1). The subjects were divided into two groups according to their characteristics: an experimental group (EG; n=11) and a control group (CG; n=9). Both groups, EG and CG, performed the AAP before the pre-tests. During the MTP, the CG followed their regular tennis training. All of the tennis-training programs (EG and CG group) were controlled and matched by volume (90 min per session, 4 sessions a week) and intensity (average sessions between 75%–85% of the individual heart rate reserve (HRR)). The MTP was included at the end of the tennis training sessions and consisted of 12 sessions (2 sessions a week; Tuesdays and Thursdays) of ∼30 min. Because the subjects were coming from tennis training, they only performed a specific warm-up, including two main exercises: supine bench press with free weights and parallel half squat using a Smith machine. The relative intensity (∼60% of 1 repetition maximum (1RM)) and rest periods (3 min) between sets were constant during the program. The number of sets increased from 3 to 6 during the MTP, with a volume decrease in the 3^rd^ and 6^th^ week (i.e., 50% and 40%, respectively). The number of repetitions per set was individually adjusted and did not change throughout the MTP, because the aim was to maintain mechanical power for the entire training session.

### Participants

A total of 20 competitive youth male tennis players (age: 15.0 ± 1 years, body height: 170.9 ± 5.1 cm, body mass: 63.3 ± 9.1 kg and 18.3 ± 6.0% body fat) involved in regular tennis competition at the national level (i.e., national ranking between 150 and 250) volunteered to take part in the study. The mean training background of the players was ∼ 5 years, which focused on tennis-specific training (i.e., technical and tactical skills) and aerobic and anaerobic training (i.e., on-and off-court exercises). Players had no regular experience in strength training, with partial experience (i.e., familiarisation sessions) in a variety of plyometric (e.g., medicine ball, hopping) and injury-prevention exercises (e.g., elastic tubing and core training). Before participation, the experimental procedures and potential risks were explained fully to the subjects, and written informed consent was obtained from the players and their parents. The study was approved by the institutional review committee of Miguel Hernández University (Elche, Spain), and it conformed to the recommendations of the Declaration of Helsinki.

### Procedures

#### Jump Tests

Squat jumps (SJ) and Countermovement jumps (CMJ) were performed on a contact platform (Globus, Italy), in accordance with Bosco et al. (1983). Each player performed 3 maximal jumps interspersed with approximately 30 s of passive recovery, and the greatest height for each jump was recorded.

#### Side medicine ball throw

Players performed a forehand and backhand medicine ball throw according to previously established methods (Roetert and Ellenbecker, 2007). Players stood sideways to the starting line and simulated a forehand/backhand stroke, tossing a 3 kg ball as far as possible, with the back leg in contact with the ground, and without crossing the line after the throw. The distance from the line to the point where the ball landed was measured, and the best performance of three trials was recorded to the nearest 5 cm.

#### Supine bench press and parallel half-squat muscular performance

Lower and upper body maximal strength was assessed using the estimated 1RM bench press and parallel half-squat actions, and was calculated using the Brzycki (1993) 1RM formula (RM = W/ [102.78–2.78(R)]/100; W = weight used; R = maximal number of repetitions performed). Subjects performed a warm-up set of 10 repetitions at 40–60% of the perceived maximum intensity. Three to six subsequent attempts were then made to determine the 1RM. Subjects were allowed to perform a maximum of 8 repetitions during the bench press and parallel half squat. Three to five min rest periods were used between lifts to ensure optimal recovery (Mayhew et al., 1995). For the supine bench press, the test began with the subject lowering the barbell from a fully extended arm position above the chest until the barbell was positioned 1 cm above the subject’s chest. From that position (supported by the bottom stops of the measurement device), the subject was instructed to perform a purely concentric action (as fast as possible) maintaining a shoulder position of 90° abduction position. This completed a successful repetition. No bouncing or arching of the back was allowed. For the parallel half squat, the subjects began with the barbell on the shoulders with the knees and hips in the extended position. As the top of the thigh reached a position parallel to the floor, and after the verbal command “up”, the subject ascended (as fast as possible) to a full knee extension of 180°. This test was performed using a Smith machine in which the barbell was attached at both ends with linear bearings allowing only vertical movements (Izquierdo et al., 2006).

Power output (i.e., leg and arm extensor muscles) was measured in the concentric portion actions of both exercises using a relative load of 60% of 1RM (W-SQUAT and W-PRESS). Two testing trials were performed, and the best result was recorded for further analyses. Moreover, an endurance test in which each subject performed maximal repetitions to failure with a load of 60% of 1RM was performed, for both bench press and parallel half squat (END-PRESS and END-SQUAT, respectively). During both exercises, barbell displacement, peak and average velocity (m·s^−1^), peak acceleration (m·s·s^−1^), peak and average force (N), and peak and average power (W) were recorded by linking a rotary encoder to the end of the barbell (T-Force Dynamic Measurement System, Ergotech©, Spain), which recorded the position and direction of the barbell. The mean relative error in the velocity measurements was found to be < 0.25%, whereas displacement was accurate to ± 0.5 mm (Sanchez-Medina and Gonzalez-Badillo, 2011).

The criterion for not leading to failure during exercise execution in MTP was to identify a significant decrease in movement velocity relative to the average velocity obtained within the first 2–3 repetitions in the endurance test (Izquierdo et al., 2006). The maximum power and average power of the best 3 repetitions were recorded. All of the data obtained from the rotary encoder were processed with customised software (Ergotech© Consulting, Spain).

#### Salivary Cortisol Samples

Three saliva samples were collected on Sundays for 11 weeks at 8 a.m., 11 a.m. and 6 p.m.. Participants provided 5–10 ml of saliva in a plastic tube with cotton (Salivette®, Sarstedt, France). Participants were instructed to complete sampling before eating or drinking. Also, participants were told to thoroughly rinse their mouths with tap water before sampling, and they were instructed not to brush their teeth before completing the saliva sampling in order to avoid the contamination of the saliva with blood caused by microinjuries in the oral cavity (Filaire et al., 2009). The samples were then collected and frozen in the laboratory’s refrigerator at −20°C until the assay. SC concentration was determined by Enzyme-Linked Immuno Sorbent Assay (ELISA) with a lower limit of sensitivity of 0.0537 μg/dl, and average intra- and inter-assay coefficients of variations (CVs) of 2.61% and 7.47%, respectively.

#### Profile of Mood States scores

The tension, depression, anger, vigour, fatigue and confusion subscales of the Spanish version of the Profile of Mood States (POMS) questionnaire were used to evaluate exercise-related mental fatigue before and after the training intervention. Total mood disturbance (TMD) was calculated using the following formula: TMD = ((Anger + Confusion + Depression + Fatigue + Tension) − Vigour) + 100. The test was administered to all participants every Sunday at 11 a.m. by the same trained interviewer.

### Statistical Analyses

Standard statistical methods were used for the calculation of means ± SD. Changes in kinematic variables were analysed with a 2-way interaction (time × group), with a series of repeated measures ANOVA, with time (T1 and T2) as the within-subjects factor, and a group (two levels: NFG, CG) as the between-subjects factor. Changes in the psychophysiological variables were analysed by a 2-way interaction (time × group) with a series of repeated measures ANOVA, with time (eight levels: baseline, six weeks intervention period, post intervention) as the within-subjects factor, and a group (two levels: NFG, CG) as the between-subjects factor. When a significant difference was found for either main effect (time or group), a Bonferroni post-hoc analysis was performed. SPSS V.20 was used for the statistical calculations. Effect sizes were calculated and interpreted according to > 0.2 (small), 0.5 (moderate) and > 0.8 (large). Statistical significance was set at the level of p < 0.05.

## Results

The players’ performance values (SJ, CMJ, side medicine ball throws, END-SQUAT, W-SQUAT, END PRESS and W-PRESS), which were obtained during T1 and T2, are presented in Tables 1 and 2. After the intervention (T2), the results showed significant improvements in the SJ (p = 0.002; η^2^ = 0.54), CMJ (p = 0.041; η^2^ = 0.34), medicine side ball throw (p = 0.001; η^2^ = 0.49), mean force (p = 0.001; η^2^ = 0.51), power (p = 0.008; η^2^ = 0.42) and velocity (p = 0.026; η^2^ = 0.36) in W-SQUAT and total number of repetitions (p = 0.001; η^2^ = 0.73), peak power (p = 0.001; η^2^ = 0.60), and mean power of the first 3 repetitions (p = 0.001; η^2^ = 0.58) in END-SQUAT for the EG, while there were no differences between the pre and post-tests in the CG for any of the variables analysed. The results also showed significant differences between the groups after T2 in mean velocity (p = 0.033; η^2^ = 0.25) during W-SQUAT, total repetitions (p = 0.001; η^2^ = 0.53), peak power (p = 0.023; η^2^ = 0.28) and mean power (p = 0.020; η^2^ = 0.29) in END-SQUAT.

No significant variations were observed in the bench press test for either group, with a non-significant increment (p = 0.079; η^2^ = 0.18) in mean power for the EG (10.5%), compared with a decrease in the CG (−4.1%) at T2.

**Figure 2:**
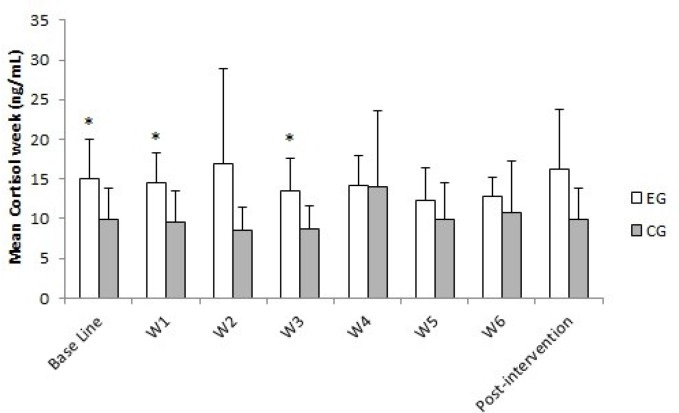
Mean values of saliva cortisol concentrations during the intervention period. ^*^Significant differences in the CG. p < 0.05.

**Figure 3:**
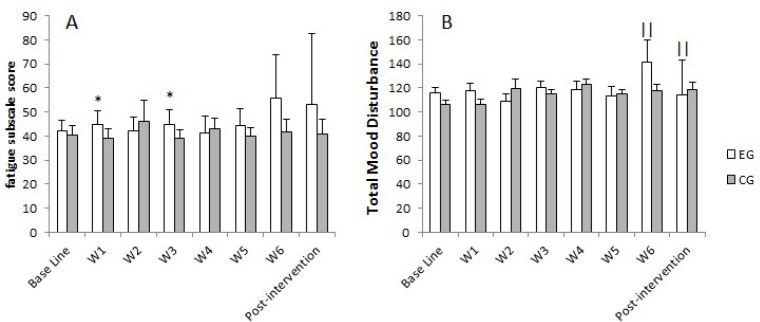
(a) Fatigue subscale score POMS. Total mood disturbance during the intervention period (b). ^*^Significant differences in the CG. p < 0.05; ^‖^Significant differences between weeks. p < 0.01

SC results showed significant differences between the groups in average baseline values (p = 0.038; η^2^ = 0.24) in week 1 (p = 0.016; η^2^ = 0.31) and 3 (p = 0.020; η^2^ = 0.30) of MTP (Figure 2). Significant differences between the groups were also observed for the fatigue subscale in week 1 (p = 0.041; η^2^ = 0.24) and 3 (p = 0.029; η^2^ = 0.26) of MTP (Figure 3a), while a significant decrease in TMD was observed between week 6 of MTP and post-intervention for the EG (p = 0.041; η^2^ = 0.48) (Figure 3b). The mean SC concentrations and the POMS scores for the EG and CG during the whole training period are presented in Figure 3.

## Discussion

The main findings of the present study were that, after a short-term strength training program not leading to muscular failure, there were improvements in the power performance (i.e., jumps, medicine ball throws and leg squats) of youth tennis players, with a moderate impact on their psychophysiological stress (i.e., small increases in cortisol, only in the first half of the training period, and small changes in the mood state, only during the tapering week).

After a 6-week strength training program not leading to failure, performance was improved in the parallel half squat, SJ, CMJ and side medicine ball throw. The results showed improvements in muscular power and the maximal number of repetitions of 8.5% and 59.2%, respectively, which are consistent with the data reported by Izquierdo et al. (2006), who found greater gains in muscular power (∼29%) and in the maximal number of repetitions performed during the parallel half squat (∼69%) when training not leading to muscular failure was performed.

With regard to the adaptations to strength and power training, lack of changes in athletes’ body mass (EG: 65.50 ± 6.87 and 65.99 ± 6.54 kg for T1 and T2, respectively) or the BMI (EG: 22.17 ± 2.30 and 22.29 ± 2.34 for T1 and T2, respectively) suggests that intrinsic muscular adaptations, motor coordination and neuromuscular activation are possible mechanisms for enhanced strength in the present study (Guy and Micheli, 2001). It is well known that neural adaptations dominate in the early stages of strength training programs, especially in youth and inexperienced athletes (Guy and Micheli, 2001). Moreover, these changes would be related to a better synchronisation of body segments and the related increased levels of motor coordination (Falk and Eliakim, 2003), supported by the significant improvements achieved in the EG in the jump tests in T2, with increases in the SJ and CMJ of 9.6% and 4.1%, respectively.

Regarding the training volume used in the present study, comparisons are difficult due to lack of studies focusing on this topic in tennis. The results are, however, in agreement with previous research reporting improvements in performance using moderate, rather than low or high volumes (González-Badillo et al., 2005) in young lifters, what suggests that training using high volumes does not seem to be necessary in order to achieve optimal strength improvements. In this regard, the training volume performed by the tennis players (∼400 repetitions in 6 weeks) was relatively low compared to the study conducted by González-Badillo et al. (2005) (∼2500 repetitions during 10 weeks), highlighting that, in a sport like tennis, in which strength demands are not maximal (Reid and Schneiker, 2008), and especially with youth athletes, training programs not leading to failure and without decreases in maximum execution velocity are effective for improving muscular strength and power. Moreover, it has been reported that strength training programs based on a high volume and leading to failure could induce overuse injuries and lead to overtraining situations (Willardson, 2007). This would be especially important in a sport like tennis, in which busy schedules limit the number of training sessions devoted to fitness development, especially during the competitive season. Moreover, in youth tennis players, we should give special care to situations in which a high frequency of specific training, combined with other activities, could increase the risk of injury (Kibler and Safran, 2005). During the past few years, it has been observed that tennis players devote a great amount of time to improve their tennis skills through technical and tactical training, with an average of 15–20 h of technical training per week, even at a young age (Crespo and Miley, 1998). As a consequence, training strategies aiming for short-term fitness improvements through a reduced number of weekly training interventions, like the one presented here, are warranted.

In contrast to the strength levels found in the lower body, upper body strength improvements were not significant. This is related to the use of free weights, which could produce greater co-activation in muscles involved in stabilising the shoulder joint during the bench press exercise, reducing the generated force due to the antagonist activation effect (Schick et al., 2010; Schwanbeck et al., 2009). Nevertheless, we found improvements in medicine ball throw performance for both the dominant and non-dominant sides of 11.6% and 8%, respectively. As previously suggested, the better synchronisation of body segments (i.e., transfer from the lower to the upper body) and the related increased levels of motor coordination produced by the training intervention could be the mechanisms responsible for these changes.

Taking into consideration the psychophysiological responses, the present training intervention produced slight effects on the neuroendocrine system (SC) and mood state (POMS). In this regard, the results are consistent with previous research showing that a high strength training volume can stimulate large secretions of SC (Nunes et al., 2011). On the other hand, and according to the criteria suggested by previous authors (Berglund and Safstrom, 1994) to identify the risk of developing chronic fatigue, the training intervention used in the present study induced low levels of psychophysiological stress. However, caution should be used in interpreting the present data, because of the great inter-individual variability in SC baseline values (Salvador et al., 2003).

SC and fatigue subscale values showed that the largest variations occurred during the first three weeks of MTP (W1 and W3). This could be related to the increase (W1) and decrease (W3) in the training load and suggests a delayed and cumulative effect of fatigue. Nevertheless, the increase in the training load (i.e., 16.7% per week) did not produce a psychophysiological (i.e., SC and POMS) impairment in the EG, compared to the CG, even during W5, in which peak training volume was achieved. Lack of changes in these variables suggests a balance between the training program conducted during our study and the subjects’ responses, which can be useful for non-experienced athletes (Faigenbaum et al., 2009). Moreover, the TMD results of the EG showed a significant decrease at the end of the training period (i.e., from W6 to post-intervention), with values returning to baseline levels, suggesting a positive adaptation to the training program and the sensitivity of the subscale fatigue for changes in the training volume (Leite et al., 2011).

It can be concluded that a non-failure strength-training protocol improved power output with a moderate psychophysiological impact on youth elite tennis players, suggesting that it is a suitable program to improve strength without large increases in total demands for these athletes. Moreover, we may consider this strength-training methodology as a good option for initial phases for non-experienced athletes.

## Figures and Tables

**Table 1:** Mean ± SD values of upper body strength tests performed during T1 and T2.

			*T1*	*T2*	*ES (η^2^)*
Side Ball throw	Dominant side (m)	EG	9.37 ± 1.01	10.60 ± 1.01 ^‡‡^	0.49
		CG	9.51 ± 1.56	10.07 ± 1.71	0.18
	Non-dominant side (m)	EG	9.07 ± 0.78	9.86 ± 1.06 ^‡^	0.42
		CG	9.46 ± 1.56	9.72 ± 1.40	0.06

W-PRESS	Velocity (m·s^−1^)	EG	0.55 ± 0.09	0.58 ± 0.08	0.09
		CG	0.61 ± 0.14	0.62 ± 0.13	0.01
	Force (N)	EG	328.0 ± 41.8	341.1 ± 48.8	0.30
		CG	285.2 ± 68.6	294.7 ± 68.7	0.37
	Power (W)	EG	182.1 ± 41.8	194.1 ± 39.6	0.14
		CG	170.2 ± 47.8	184.2 ± 74.8	0.09

END-PRESS	Rep. until failure	EG	15.0 ± 5.6	23.7 ± 9.4	0.56
		CG	8.6 ± 6.0	8.3 ± 2.4	0.41
	Rep. not leading to failure	EG	7.7 ± 3.2	11.0 ± 5.6	0.50
		CG	6.4 ± 5.9	6.7 ± 1.8	0.11
	Peak power (N)	EG	383.0 ± 104.4	514.7 ± 124.2	0.29
		CG	360.7 ± 105.7	379.1 ± 86.9	0.50
	Mean power (N)	EG	375.1 ± 102.5	500.4 ± 118.4	0.28
		CG	345.5 ± 103.2	368.1 ± 82.9	0.54

W-PRESS = Bench press power output test;

END-PRESS= Bench press endurance test.

‡ Significant differences from T1. p < 0.05;

‡‡ Significant differences from T1. p < 0.01

**Table 2:** Mean ± SD values of lower limb tests performed during T1 and T2, and effect sizes (ES)

			T1	T2	ES (η^2^)
Jump Tests	SJ (cm)	EG	28.45 ± 3.61	31.18 ± 2.27 ^‡‡^	0.54
		CG	31.71 ± 4.68	33.28 ± 3.59	0.38
	CMJ (cm)	EG	31.18 ± 3.57	32.45 ± 2.33 ^‡^	0.34
		CG	33.85 ± 3.57	33.57 ± 4.46	0.02

W-SQUAT	Velocity (m·s^−1^)	EG	0.57 ± 0.09	0.62 ± 0.13 ^*‡^	0.36
		CG	0.55 ± 0.02	0.50 ± 0.06	0.40
	Force (N)	EG	627.9 ± 183.1	685.1 ± 181.8 ^‡‡^	0.51
		CG	700.8 ± 231.0	700.1 ± 231.4	0.23
	Power (W)	EG	351.6 ± 91.8	405.0 ± 105.2 ^‡‡^	0.42
		CG	380.8 ± 117.1	347.7 ± 111.4	0.38

END-SQUAT	Rep. until failure	EG	14.9 ± 5.6 ^*^	23.73 ± 9.36 ^**‡‡^	0.73
		CG	8.6 ± 6.0	8.29 ± 2.43	0.01
	Rep. not leading to failure	EG	7.6 ± 3.2	11.0 ± 5.6	0.37
		CG	6.4 ± 5.9	6.7 ± 1.8	0.01
	Peak power (N)	EG	383.0 ± 104.4	514.7 ± 124.2 ^*‡‡^	0.60
		CG	360.7 ± 105.7	379.1 ± 86.9	0.07
	Mean power (N)	EG	375.1 ± 102.5	500.4 ± 118.3 ^*‡‡^	0.58
		CG	345.5 ± 103.2	368.1 ± 81.9	0.11

SJ = Squat jump; CMJ = Countermovement Jump;

W-SQUAT = Parallel half-squat power output test;

END-SQUAT= Parallel half-squat endurance test.

‡ Significant differences from T1. p < 0.05;

‡‡ Significant differences from T1. p < 0.01;

* Significant differences in the CG. p < 0.05;

** Significant differences in the CG. p < 0.01
